# Serologic and Molecular Biologic Methods for SARS-associated Coronavirus Infection, Taiwan

**DOI:** 10.3201/eid1002.030731

**Published:** 2004-02

**Authors:** Ho-Sheng Wu, Shu-Chun Chiu, Tsan-Chang Tseng, Szu-Fong Lin, Jih-Hui Lin, Yu-Fen Hsu, Mei-Ching Wang, Tsuey-Li Lin, Wen-Zieh Yang, Tian-Lin Ferng, Kai-Hung Huang, Li-Ching Hsu, Li-Li Lee, Jyh-Yuan Yang, Hour-Young Chen, Shun-Pi Su, Shih-Yan Yang, Ting-Hsiang Lin, Ih-Jen Su

**Affiliations:** * Center for Disease Control, Department of Health, Taiwan, Republic of China

**Keywords:** SARS-CoV, serodiagnostic methods, neutralization test, IFA, ELISA, immunochromatographic test, Western blot

## Abstract

Severe acute respiratory syndrome (SARS) has raised a global alert since March 2003. After its causative agent, SARS-associated coronavirus (SARS-CoV), was confirmed, laboratory methods, including virus isolation, reverse transcriptase–polymerase chain reaction (RT-PCR), and serologic methods, have been quickly developed. In this study, we evaluated four serologic tests ( neutralization test, enzyme-linked immunosorbent assay [ELISA], immunofluorescent assay [IFA], and immunochromatographic test [ICT]) for detecting antibodies to SARS-CoV in sera of 537 probable SARS case-patients with correlation to the RT-PCR . With the neutralization test as a reference method, the sensitivity, specificity, positive predictive value, and negative predictive value were 98.2%, 98.7%, 98.7%, and 98.4% for ELISA; 99.1%, 87.8%, 88.1% and 99.1% for IFA; 33.6%, 98.2%, 95.7%, and 56.1% for ICT, respectively. We also compared the recombinant-based western blot with the whole virus–based IFA and ELISA; the data showed a high correlation between these methods, with an overall agreement of >90%. Our results provide a systematic analysis of serologic and molecular methods for evaluating SARS-CoV infection.

Severe acute respiratory syndrome (SARS) is a new infectious disease with clinical symptoms indistinguishable from atypical pneumonia at the early stage of illness (*1*). Because of its relatively high transmissibility and mortality rate on infection, >8,400 SARS patients, including 810 deaths, have been reported by China, Vietnam, Hong Kong, Singapore, Canada, Taiwan, and other areas worldwide from March to July 2003 (*2*). As of July 31, 668 probable SARS case-patients, including 71 deaths, were reported to the Center for Disease Control, Taiwan (Center for Disease Control-Taiwan) (*3*). With the close cooperation of laboratories worldwide, the causative agent of SARS was quickly identified as a new coronavirus species, now referred to as SARS-associated coronavirus (SARS-CoV) (*4*–*6*). With epidemiologic evidence, droplet and close contact transmission are the major routes for the spread of SARS (*7*). Suspected SARS patients need to be quarantined and treated with intense care to minimize transmission to others. Therefore, sensitive and specific laboratory tests to differentiate SARS from other mild atypical pneumonia must be developed to shorten the quarantine period for contacts with SARS patients and further to contain SARS outbreaks.

Even though the RT-PCR is the most sensitive technique to detect early SARS-CoV infection, the positive predictive rate for probable SARS cases is only 37.5% according to our data (Center for Disease Control-Taiwan). The other reported probable SARS cases, therefore, still have to rely on serologic diagnosis. We analyzed the results from immunofluorescence assay (IFA), enzyme-linked immunosorbent assay (ELISA), neutralization test, and immunochromatographic test (ICT) to detect antibodies against SARS-CoV in serum specimens of patients with probable SARS in Taiwan. The results of neutralization tests, ELISA, and IFA were highly correlated.

## Materials and Methods

### Specimens

According to World Health Organization (WHO) criteria, a person seeking treatment after November 1, 2002, with a history of high fever (>38°C), coughing, or breathing difficulty, and having resided in or traveled to an area with recent local transmission of SARS during the 10 days before onset of symptoms was classified as a suspected case-patient. A suspected case-patient with radiographic evidence of infiltrates consistent with pneumonia or respiratory distress syndrome on a chest x-ray was considered a probable case-patient (*8*). In the study, 3,367 throat swab specimens from possible SARS patients reported to Center for Disease Control-Taiwan were tested for SARS-CoV by RT-PCR. Seven hundred and ninety-nine serum samples from 537 probable case-patients, fulfilling WHO criteria for probable SARS cases, were tested for antibodies to SARS-CoV by neutralization test, IFA, ELISA, and ICT. Of these patients, 262 had paired serum specimens, in which the acute- and convalescent-phase serum specimens were collected at day 1 to day 12 and at day 28 or more after the onset of illness, respectively. In the other 275 patients, only a single serum specimen was collected during their illness: 210 had the serum collected at the acute phase or at the early convalescent phase from day 1 to day 20, and 65 were collected during the late convalescent phase from day 28 to day 78 after the illness onset.

### RT-PCR

The primers and probes used for SARS-CoV detection by RT-PCR and real-time RT-PCR were synthesized, according to the recommendations of the Centers for Disease Control and Prevention (CDC), Atlanta, Georgia, USA (*5*,*9*). The viral RNA from the throat swab specimens was extracted by the MagNA LC Pure and MagNA Pure LC total nucleic acid isolation kit (Rouche, Mannheim, Germany). After extraction, 5 μL of RNA extract was used as the template in all PCR assays in 50-μL reaction volumes containing 10 μL of 5X buffer, 2 μL enzyme mix, 2 μL deoxynucleoside triphosphate (dNTP), and 0.6 μM each of sense and antisense primer. The reaction was subjected to precycle condition at 50°C for 30 min, and 95°C for 15 min. Forty cycles of amplification were then conducted at 95°C for 30 s, 50°C for 40 s, and 72°C for 1 min. For real-time quantitative RT-PCR assays, a 20-μL reaction volumes containing 12 μL of HPA (human pneumonia–associated coronavirus)-Coronavirus LC Master mix, 3 μL of HPA-Coronavirus LC Mg-Sol, and 0.5 μL of HPA-Coronavirus LC internal control were thermal-cycled by a Light Cycler (Rouche) at 50°C, for 10 min for RT reaction, at 95°C for 10 min for denaturation, and followed by 45 cycles of amplification at 95°C for 2 s, 55°C for 12 s, and 72°C for 10 s.

### Neutralization Test

Serum specimens were tested for neutralizing activity, according to the procedures described by Marx et al. (*10*), with modifications. The neutralization titer was determined in Vero E6 cells. Briefly, the serum specimens from patients with probable SARS were first incubated at 56°C for 30 min. Then, 50 μL of serial twofold diluted serum specimen, from 8-fold to 1,024-fold were added into equal volume of culture medium containing SARS-CoV (50 tissue culture infective dose [TCID_50_] on a 96-well microtiter plate and incubated at 37°C for 1 h. Finally, 100 μL of Vero E6 cells (2.5 x 10^5^/μL) were added to each well of the plate. Cultures were held at 37°C and 5% CO_2_ with daily observations for cytopathic effect (CPE). On day 5, the titer of antibody was calculated as the highest dilution that CPE was completely inhibited on the well. The neutralization test was carried out with each sample in duplicate along with both positive and negative controls. The positive control serum specimens were taken from patients with confirmed SARS in Taiwan, and the negative control serum specimens were from healthy volunteers. If a sample showed a 4-fold difference or greater in titers in the duplicated sample runs, it was judged as an invalid outcome and had to be retested. A sample is considered to be positive if its titer is >1:16 in the case of single serum group, and at least a 4-fold increase in titers between the acute- and convalescent-phase serum specimens in the paired specimens group.

### IFA

IFA testing was performed by using a diluted serum specimen reacted against SARS-CoV-infected Vero E6cells and uninfected Vero E6cells. Vero E6 cells were grown in minimum essential medium (MEM) containing 10% fetal bovine serum at 37°C. At a density of 80%, the cells were infected with SARS-CoV (TCID_50_, 10^6^/mL). After CPE appeared, the cells were washed with 0.025% trypsin and spotted on slides for IFA as previously described (*11*). These slides were put in a closed heating container until completely drying, then were fixed in acetone for 15 min. 10 μL of 2-fold serial diluted serum starting from >1:100 to 1:800 was placed onto each well of the slide, and incubated at 37°C for 30 min. After being washed twice with phosphate-buffered saline (PBS), for 5 min each, 10 μL of 1:100 diluted specific antihuman gamma globulins labeled with FITC (Zymed) was added onto each well, and incubated at 37°C for 30 min. After washing twice with PBS, slides were observed under a fluorescence microscope. Criteria for a positive IFA result included reactivity to infected cells. A sample with an antibody titer of 1:100 is positive. Sera that did not react to infected cells were considered negative. If nonspecific reactivity to both infected and uninfected cells were detected, the test was considered un-interpretable.

### ELISA

An ELISA for the detection of coronavirus has been described (*12*). In our study, the materials for the ELISA to detect SARS-CoV antibodies were provided by CDC in Atlanta. In brief, SARS-CoV Vero E6 cell lysates used as antigens were added to the top half of the wells in the plate overnight at 4°C. The Vero E6 cell lysates without SARS-CoV used as control antigens were simultaneously added to the wells in the bottom half of the plate. On the following day, 100 μL of diluted serum (starting from 1:100 to 1:1,600) was added to both test and control wells. Then each well of the plate was incubated at 37°C for 60 min. After washing the plate 3 times with 250 μL of wash buffer in each well, add 100 μL of conjugate dilution (1:4,000 of goat anti-human immunoglobulin (Ig)A, IgG, and IgM) to each well and incubate the plate at 37°C for 60 min. Again after washing, 100 μL of the substrate (a 1:1 mixture of 2,2-azino-di [3-ethylbenzthiazoline] sulfonic acid [ABTS] and hydrogen peroxide) were added to each wells, and incubated at 37°C for 30 min. Place the plate on ELISA reader, and read at 410 nm. A sample is positive if its adjusted optical density (OD) value (OD of test – OD of control) exceeds the mean plus 3 standard deviations of the normal controls and its titer is >1:400.

### ICT

The ICT generally refers to a rapid chromatographic technique based on a sandwich format using double antigens or double antibodies (*13*). The SARS-CoV rapid test we adopted is a newly developed immunogold-based ICT device (Tyson Bioresearch, Inc.,Taipei, Taiwan). The antigen used in this test is a recombinant nucleocapsid (N) protein of SARS-CoV. The inside of the ICT device contains a nitrocellulose strip, on top of which is a detection zone. In the detection zone, the goat anti-mouse IgG and SARS-CoV N protein have been immobilized separately onto a control line and a test line. In the middle of the strip, the mouse IgG and SARS-CoV N protein are to be coupled respectively with some colloidal gold particles, which serve as a detector. At the bottom are two wells for the sample and the buffer, respectively. The ICT is carried out following the manufacture’s instruction. Briefly, 15 μL of undiluted serum sample is added to the sample well, and 220 μL of testing buffer to the buffer well. When the sample contains specific antibodies to SARS-CoV, they will react first with the antigen-gold complex. After lateral flow along the membrane, a colored complex of antibodies-antigen-gold will deposit on the test line containing the fixed antigen. The red signal from the gold will gradually appear on the test line and become visible by naked eye. A positive result will show two parallel lines; the upper one is the control line, which shows that the device works fine and the lower one is the test line, which indicates that the serum sample contains SARS-CoV antibodies. In case of a negative result, only red will be seen on the control line. If red is found only at the test line or no lines are visible, the test is invalid.

### Western Blot

The preparation of recombinant proteins of SARS-CoV and the procedures for Western blot assay have been described recently (*14*). Briefly, the amplified gene products of SARS-CoV including N, M (membrane), and S (spike), were gel purified and cloned into the pQE30 expression vector (Qiagen, Valancia, CA). The constructs were then transformed into *Escherichia coli* JM109 cells (Invitrogen, Carlsbad, CA). After induction by isopropyl-β-D-thiogalactopyranoside, the cells were sonicated, and the recombinant proteins were extracted with 1.5% sarcosine. Finally these recombinant proteins were bound by BD TALON metal affinity resins (BD Biosciences, San Jose, CA) and examined by 12% sodium dodecyl sulfate–polyacrylamide gel electrophoresis. The Western blot assay was carried out to examine the pattern of antibody development against different recombinant proteins of SARS-CoV.

## Results

### Detection of Viral RNA of SARS-CoV by RT-PCR

**Figure 1:**
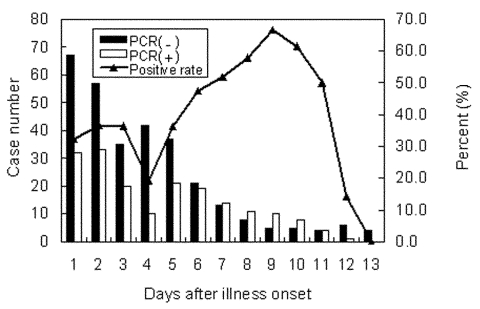
Polymerase chain reaction–positive rates of throat swab specimens collected on different days from probable SARS cases. If a patient had two or more specimens, the patient was only counted once.

A total of 3,367 possible SARS patients were reported to Center for Disease Control-Taiwan from March 10 through the end of July 2003. Of which, 668 were probable case-patients, 1,331 were suspected case-patients, 1,036 were rejected, and 332 case-patients were removed from reporting (Table 1). Throat swabs were collected from 590 of the 668 patients with probable cases. Of them, 221 had positive results on PCR, giving a positive rate of 37.5%. Throat swabs were also collected from 1,043 of the 1,331 patients with suspected cases. Of them, 38 had positive results by PCR, giving a positive rate of 3.6%. Figure 1 shows the PCR-positive rates of the throat swab specimens taken from patients with probable SARS between day 1 and day 13 after the illness onset. On the first day of onset, RT-PCR detected positive results in 32% of patients with suspected cases. The positive rates reached a peak of 50% to 60% on day 7 to day 10 and declined thereafter. However in a few specimens, virus RNA was still detected on day 18, day 20, and day 38 after illness onset (data not shown).

**Table 1:** Positive rates of RT-PCR for SARS-CoV in reported SARS cases in Taiwan

Classification of reported cases	Case no.	Specimens collected^a^	No. PCR (+)	Positive rate (%)
Probable	668	590	221	37.5
Suspected	1,331	1,043	38	3.6
Ruled out	1,036	907	7	0.8
Reporting cancelled	332	229	1	0.4
Total	3,367	2,769	267	9.6

^a^Throat swab specimens were used for RT-PCR (reverse transcriptase–polymerase chain reaction). SARS-CoV, severe acute respiratory syndrome–associated coronavirus.

### Detection of Antibodies to SARS-CoV in Probable SARS Patients

**Figure 2:**
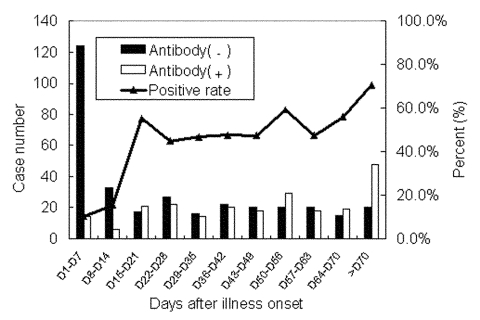
Antibody positive rate of serum specimens collected on different days from probable SARS case-patients. If a patient had two or more specimens, the patient was only counted once.

Figure 2 shows when antibodies to SARS-CoV appeared during the infection. Although in samples from 10% (14/138) of the probable case-patients, antibodies to SARS-CoV could be detected during the acute phase of illness (day 1 to day 7) by Nt, IFA, or ELISA, antibodies against SARS-CoV developed in most at the late convalescent stage. The positive rate of antibodies to SARS-CoV was raised to 50% at 3 weeks after illness onset and reached to a peak of over 70% at 10 weeks after onset. The overall antibody-positive rate was 54.2% (254/469).

### Relative Values of Different Serodiagnostic Methods

Of the total 537 probable SARS case-patients, 469 had been tested for the antibody response to SARS-CoV by neutralization test, ELISA, and IFA in parallel, but only 244 patients were tested by ICT. With neutralization tests as a reference method, the overall characteristics of the evaluated methods, including ELISA, IFA, and ICT, are given in Table 2. For ELISA, the sensitivity was measured at 98.2%. Of the 224 serum specimens, which tested positive with neutralization test, 4 gave negative responses with ELISA. The specificity, positive predictive value, and negative predictive value were 98.7%, 98.7%, and 98.4%, respectively. For IFA, the sensitivity was evaluated at 99.1%. Two serum samples, which had been positive in neutralization test, were negative with IFA. The specificity, positive predictive value, and negative predictive value were of 87.8%, 88.1%, and 99.1%, respectively. The specificity of the ICT was calculated to be 98.2%; however, its sensitivity (33.6%) was low, leading to a negative predictive value of 56.1%. In the total of 245 negative neutralization tests were 3 positive results were detected with ELISA, 30 positive with IFA, and 2 positive with ICT tests. These 35 specimens were taken from 31 patients, in which two positive PCR results were found.

**Table 2:** Specificity, sensitivity, positive and negative predictive values of the tests evaluated for the serodiagnosis of SARS, in comparison to the neutralization test^a,b^

			Neutralization test		Performances of methods evaluated			
Method	Results	Number	Positive	Negative	Sensitivity	PPV	Specificity	NPV
								
ELISA	Positive	223	220	3	98.2%	98.7%	98.7%	98.4%
	Negative	246	4	242				
IFA	Positive	252	222	30	99.1%	88.1%	87.8%	99.1%
	Negative	217	2	215				
ICT^c^	Positive	46	44	2	33.6%	95.7%	98.2%	56.1%
	Negative	198	87	111				

### Cross-Reactions with the Non-SARS Panel

Ten normal serum samples from healthy volunteers tested negative for antibodies against SARS-CoV by neutralization test, IFA, ELISA, and ICT. In addition, 24 serum samples from patients with other diseases were used as a specificity panel to analyze whether these assays showed any cross reactions with SARS-CoV. These patients were definitely confirmed as non-SARS-CoV–associated diseases. As shown in Table 3, no positive results were detected to these serum specimens, and the measurements of specificity were all 100% for the neutralization test, ELISA, IFA, and ICT.

**Table 3:** Specificity of the tests evaluated for the serodiagnosis of SARS, in comparison to the neutralization test with regards to samples which tested positive for other diseases^a,b^

			Neutralization test		ELISA		IFA		ICT	
Pathogen	Parameter	Number	Positive	Negative	Positive	Specificity	Positive	Specificity	Positive	Specificity
Hepatitis B virus	HBs IgM	3	0	3	0	100%	0	100%	0	100%
Hepatitis C virus	IgM	3	0	3	0	100%	0	100%	0	100%
Adenovirus	Total Ab	1	0	1	0	100%	0	100%	0	100%
Influenza A virus	Total Ab	3	0	3	0	100%	0	100%	0	100%
Influenza B virus	Total Ab	1	0	1	0	100%	0	100%	0	100%
Dengue virus	IgM	2	0	2	0	100%	0	100%	0	100%
JEV	IgM	1	0	1	0	100%	0	100%	0	100%
Hantavirus	Total Ab	1	0	1	0	100%	0	100%	0	100%
*Chlamydia pneumoniae*	IgM	4	0	4	0	100%	0	100%	0	100%
*Mycoplasma pneumoniae*	IgM	4	0	4	0	100%	0	100%	0	100%
*Streptococcus pneumoniae*	Total Ab	1	0	1	0	100%	0	100%	0	100%
Total non-SARS pathogens		24	0	24	0	100%	0	100%	0	100%

### Values of RT-PCR and Neutralization Test

Table 4 compares results of the RT-PCR and the neutralization test in specimens from probable SARS case-patients. In this comparison, throat swab specimens from 381 probable SARS case-patients were used for RT-PCR, and their convalescent-phase serum specimens, collected on day 28 or longer after illness onset, were tested with neutralization test. Of the 207 cases, which were positive by neutralization test, 145 were tested positive with RT-PCR. The sensitivity, specificity, positive predictive value, and negative predictive value of RT-PCR compared with results with neutralization test were of 52.2%, 78.7%, 74.5%, and 58.1%, respectively.

**Table 4:** Specificity, sensitivity, positive and negative predictive values of the RT-PCR for the diagnosis of SARS, in comparison to the neutralization test with convalescent-phase serum specimens^a^

			Neutralization test		Performances of methods evaluated			
Method	Results	No.	Positive	Negative	Sensitivity	PPV^b^	Specificity	NPV^c^
RT-PCR	Positive	145	108	37	52.2%	74.5%		
	Negative	236	99	137			78.7%	58.1%

### Laboratory Confirmation Rate for Probable SARS Case-Patients

Table 5 shows the laboratory confirmation rate of probable SARS cases in Taiwan. With 469 probable case-patients tested, the positive rate of RT-PCR is 33.7% (158/469). These patients had been also tested for the antibody response to SARS-CoV by neutralization test, ELISA, and IFA, but only 244 were tested by ICT. The seropositive rate for ELISA, IFA, neutralization test, and ICT were 47.5% (223/469), 57.7% (252/469), 47.8% (224/469), and 16.8% (41/224), respectively. If these results were combined with existing RT-PCR results, the laboratory confirmation rates of probable SARS cases went up to 57.4% (269/469), 63.3% (297/469), 57.8% (271/469), and 42.4% (103/244), respectively.

**Table 5:** Laboratory confirmation rate in probable SARS cases, in combination of RT-PCR with different serologic methods^a^

Results	ELISA	IFA	Neutralization test	ICT
PCR (+)	33.7% (158/469)	33.7% (158/469)	33.7% (158/469)	35.7% (87/244)
Antibody (+)	47.5% (223/469)	57.7% (252/469)	47.8% (224/469)	16.8% (41/244)
PCR (+) or antibody (+)	57.4% (269/469)	63.3% (297/469)	57.8% (271/469)	42.4% (103/244)

### Recombinant Antigens for SARS Serologic Diagnosis

As discussed above, all the neutralization tests, ELISA, and IFA are based on the whole viral extracts of SARS-CoV. Therefore, antigens for these serologic tests must be prepared in the biosafety level 3 laboratory. To provide a convenient tool and decrease the risk of infection, a Western blot with several SARS-CoV recombinant proteins was developed and evaluated. Cloned peptides carrying epitopes can be produced on a large scale and with an acceptable degree of purity. Table 6 shows the comparison of recombinant protein- based Western blot with whole virus- based IFA, and ELISA. Ninety-five serum samples were used in this comparison. The sensitivities, specificities and overall agreements of Western blot were 91.3%, 89.88%, and 90.5%, compared with IFA results; 97.6%, 88.8%, and 92.6%, compared with ELISA results.

**Table 6:** Comparison of recombinant protein–based Western blot with whole virus–based IFA and ELISA^a,b^

			IFA					ELISA				
Method	Results	Number	Positive	Negative	Sensitivity	Specificity	Overall agreement^a^	Positive	Negative	Sensitivity	Specificity	Overall agreement^a^
Western blot	Positive	47	42	5	91.3%	89.8%	90.5%			97.6%	88.8%	92.6%
	Negative	48	4	44								

## Discussion

The study shows that in the first 2-week period after onset of SARS, RT-PCR is the most sensitive method of detecting the virus RNA, and the positive rate is the highest. However, during the convalescent phase of the disease, detecting antibodies in serum specimens is more important than detecting viral RNA. Four serologic diagnostic methods, including neutralization test, ELISA, IFA, and ICT were each evaluated and compared for antibody responses to SARS-CoV infection, in which the neutralization test was held as a reference method. The specificity of these methods is extremely good (100%), since no cross- reactions were detected with a non-SARS disease panel.

However, some variations in sensitivity, positive predictive value, and negative predictive value were found among these methods. As shown in Table 2, ELISA results were highly correlated with results from the reference method, the neutralization test. The measured performance of ELISA was so outstanding, with the sensitivity, specificity, positive predictive value, and negative predictive value levels exceeding 98%, that ELISA was chosen as a confirmation alternative. In the case of IFA, both the sensitivity and negative predictive value levels were above 99%; however, the specificity of 87.8% implies that IFA may cause false-positive problems. Therefore, a weak positive IFA result should be retested by a neutralization test or ELISA. The ICT, though simple and quick to perform, is lacking in adequate sensitivity in our evaluation. Therefore, it was not a reliable test for detecting of antibodies to SARS-CoV.

Since the neutralization test, ELISA, and IFA all use whole virus particles as the antigen, for safety reasons the preparation of SARS-CoV antigen must be conducted in a biosafety level 3 laboratory, which will prevent these test methods from being widely applied. Therefore, the trend in method development may lead toward the manufacturing of antigens with certain recombinant proteins. In this study, we compared a recombinant-based Western blot with the whole virus-based IFA and ELISA, and the data showed a high degree of correlation between these methods, with an overall agreement above 90% (Table 6). Thus, using these recombinant antigens may become a much safer alternative to detect antibodies against SARS-CoV.

Eight PCR-positive specimens were found in the group of the ruled out and group of those that were reported canceled (Table 1), and they were selected to test for antibodies to SARS-CoV by using acute-phase serum samples between day 1 and day 4 after the illness onset. However, no positive result was found by any of the IFA, ELISA, and neutralization test. Since no convalescent-phase serum specimens were collected from those patients, we do not know the negative results are truly negative or just resulted from the timing of gathering specimens when no antibodies were produced. Moreover, another 95 samples from the ruled-out category had been tested with ELISA, but no positive results were found. In addition, 283 specimens from 1,036 case-patients with suspected SARS were also assayed with ELISA and the neutralization test. Of them, 45 were positive with a positive rate of 15.9% (45/283). Among the 35 PCR-positive specimens in the suspected SARS category, 10 were also positive in detection of antibodies to SARS-CoV.

Finally, in this study, the overall antibody positive rate for probable SARS patients was 54.2%. This rate was much lower than that reported in Hong Kong, which showed that the IgG seroconversion to SARS coronavirus was as high as 93% (70/75) at day 28 after the illness onset (*15*). This difference may come from some different circumstances between Hong Kong and Taiwan. In the SARS outbreak of Hong Kong, the index case-patient and the infectious source leading to the outbreak were quite clear, and 75 patients were admitted to the same hospital within 4 days. From the epidemiologic point of view, therefore, the SARS outbreak was a typical cluster outbreak. In Taiwan, the samples from probable SARS case-patients were collected from over 50 hospitals between March and June 2003. Some might not have been true SARS patients but were reported as probable SARS cases. This result is likely due to the policy that suspicious SARS cases were to be reported to local health agency within 24 hours in Taiwan or the clinician who attended the patients would have been fined. In September 2003, according to the WHO criteria and the laboratory data, 346 patients were reclassified as probable SARS patients by the Center for Disease Control-Taiwan, and these data were readily accepted by WHO on September 26, 2003 (*16*). With this new classification, the positive rate of antibodies to SARS-CoV in probable SARS patients in Taiwan was increased to 86.6% (227/262), by using the serum samples on day 28 or beyond after the onset of illness. These rates are closer to, though still lower than, rates from Hong Kong. Samples from the remaining 322 cases, excluded from the category of probable SARS cases, may have to be tested for other pathogens, such as *Mycoplasma pneumoniae, Chlamydia pneumoniae,* and human metapneumovirus to clarify a diagnosis.
